# Three Toxic Heavy Metals in Open-Angle Glaucoma with Low-Teen and High-Teen Intraocular Pressure: A Cross-Sectional Study from South Korea

**DOI:** 10.1371/journal.pone.0164983

**Published:** 2016-10-21

**Authors:** Si Hyung Lee, Eun Min Kang, Gyu Ah Kim, Seung Woo Kwak, Joon Mo Kim, Hyoung Won Bae, Gong Je Seong, Chan Yun Kim

**Affiliations:** 1 Institute of Vision Research, Department of Ophthalmology, Severance Hospital, Yonsei University, College of Medicine, Seoul, Korea; 2 Department of Ophthalmology, Eulji University Hospital, Daejeon, South Korea; 3 Department of Statistics, University of Georgia, Athens, Georgia, United States of America; 4 Department of Ophthalmology, Kangbuk Samsung Hospital, Sungkyunkwan University School of Medicine, Seoul, Republic of Korea; National Eye Institute, UNITED STATES

## Abstract

**Background:**

To investigate the association between heavy metal levels and open-angle glaucoma (OAG) with low- and high-teen baseline intraocular pressure (IOP) using a population-based study design.

**Methods:**

This cross-sectional study included 5,198 participants older than 19 years of age who participated in the Korean National Health and Nutrition Examination Survey (KNHANES) from 2008 to 2012 and had blood heavy metal levels available. The OAG with normal baseline IOP (IOP ≤ 21 mmHg) subjects were stratified into low-teen OAG (baseline IOP ≤ 15 mmHg) and high-teen OAG (15 mmHg < baseline IOP ≤ 21 mmHg), and the association between blood lead, mercury, and cadmium levels and glaucoma prevalence was assessed for low- and high-teen OAG.

**Results:**

The adjusted geometric mean of blood cadmium levels was significantly higher in subjects with low-teen OAG than that of the non-glaucomatous group (P = 0.028), whereas there were no significant differences in blood lead and mercury levels. After adjusting for potential confounders, the low-teen OAG was positively associated with log-transformed blood cadmium levels (OR, 1.41; 95% confidence interval (CI), 1.03–1.93; P = 0.026). For high-teen OAG, log-transformed blood levels of the three heavy metals were not associated with disease prevalence. The association between log-transformed blood cadmium levels and low-teen OAG was significant only in men (OR, 1.65; 95% CI, 1.10–2.48; P = 0.016), and not in women (OR, 1.10; 95% CI, 0.66–1.85; P = 0.709).

**Conclusions:**

The results of this study suggest that cadmium toxicity could play a role in glaucoma pathogenesis, particularly in men and in OAG with low-teen baseline IOP.

## Introduction

Glaucoma is among the leading causes of blindness worldwide [1], and open-angle glaucoma (OAG) is known to be the most prevalent form. In particular, normal-tension glaucoma (NTG), defined as OAG with an intraocular pressure (IOP) ≤ 21 mmHg, accounts for a significant fraction among all subtypes of glaucoma in Asian populations, especially in Korea and Japan [2–4]. Although IOP has been identified as one of the main risk factors for the development and progression of NTG, non-IOP factors have been suggested as a cause of glaucomatous optic nerve damage, particularly in patients with low-teen baseline IOP (≤15 mmHg) [5–9].

Heavy metals, ubiquitous environmental pollutants that typically have long biological half-lives in humans, have recently been suggested as risk factors for various chronic diseases, such as hypertension [10–12], diabetes mellitus [13, 14], metabolic syndrome [15, 16], cardiovascular diseases [17, 18], and neurodegenerative diseases [19]. Moreover, a number of studies have reported that higher toxic heavy metal levels are significantly related to several ocular pathologies, including age-related macular degeneration (AMD) [20, 21], cataracts [22–24], and pseudoexfoliation syndrome [25–27]. A possible relationship between abnormal toxic heavy metal levels and optic nerve damage has also been suggested. Previous studies demonstrated an association between the body burden of lead and glaucoma [28, 29], and several experimental studies revealed that cadmium exposure induced impaired neurogenesis and axonal development [30, 31]. Although the evidence indicates that toxic heavy metals might have a role in the pathogenesis of glaucoma, their association with glaucoma remains poorly understood.

Using a population-based study design, we investigated whether blood concentrations of three toxic heavy metals (lead, mercury, and cadmium) are associated with the prevalence of OAG with normal IOP (OAG with baseline IOP ≤ 21 mmHg), low-teen OAG (OAG with baseline IOP ≤ 15 mmHg) and high-teen OAG (OAG with baseline IOP 15–21 mmHg). In addition, since previous studies revealed differences in heavy metal toxicity between men and women [16, 20, 28, 32–34], we examined gender differences in the association between blood heavy metal levels and OAG prevalence.

## Methods

### Study population

The current study was based on data from the Korean National Health and Nutrition Examination Survey (KNHANES), a nationwide, population-based, cross-sectional health examination and survey conducted by the Korean Centers for Disease Control and Prevention, with approval from its Institutional Review Board (IRB).[35] The KNHANES adopted a multistage, stratified, probability-clustered sampling method. The target population of the survey included the civilian noninstitutional population of South Korea. The survey consisted of health records collected from a health interview, a health examination, and a nutrition survey. The interview included demographic, socioeconomic, health, and nutritional questions. Health examinations included vital signs, physiologic measurements, and basic laboratory tests. Ophthalmologic interview and examination data were available from the second half of 2008.

In this study, data from KNHANES IV (2008, 2009) and V (2010–2012) were used to investigate the association between blood lead, mercury, and cadmium levels and NTG. Among 45,811 participants, 32,949 individuals aged ≥19 years with ophthalmologic examination data were included for the study. For such population size, the calculated recommended sample size is 380 for 5% of margin of error and 95% confidence level. Among 32,949 participants, only a selected subpopulation underwent measurement of blood heavy metal concentrations. To provide nationally representative estimates, heavy metal measurements were performed in 10 randomly sampled participants from each district with age and sex stratification from the annual KNHANES databases. Three separate data sets were formed for heavy metals for statistical analyses. Participants with age-related macular degeneration and diabetic retinopathy were excluded, since these are the major causes of non-glaucomatous visual field defects and could result in the misdiagnosis of glaucoma.

The study design adhered to the principles outlined in the Declaration of Helsinki for research involving human subjects, and all survey participants provided written informed consent. IRB/Ethics Committee of Yonsei University Health System approval was obtained.

### Ophthalmic survey components

The ophthalmologic examination consisted of ophthalmologic interviews, visual acuity measurements, IOP measurements, auto-refraction, slit-lamp examination, and fundus photography. The slit-lamp examination was performed to detect anterior segment abnormalities and measure anterior chamber depth using the Van Herick method. The spherical equivalent was calculated as sphere + 1/2 cylinder. IOP was measured once per eye from right to left with a Goldmann applanation tonometer (Haag-Streit model BQ-900; Haag-Streit Inc., Bern, Switzerland) by a trained ophthalmologist during the slit-lamp examination. A digital non-mydriatic retinal camera (TRC-NW6S; Topcon Inc., Tokyo, Japan) and a Nikon D-80 digital camera (Nikon Inc., Tokyo, Japan) were used to obtain the digital fundus images, which were captured under physiological mydriasis. Based on the fundus images, horizontal and vertical cup-disc ratios (VCDRs) were measured, and any signs of diabetic retinopathy or age-related macular degeneration were checked.

A visual field test with frequency double technology was performed in participants who had elevated IOP (≥22 mmHg) or features indicative of glaucomatous optic disc. The indicative features of glaucomatous optic disc included: (1) horizontal or VCDR ≥ 0.5; (2) presence of optic disc hemorrhage; (3) presence of retinal nerve fiber layer defects; or (4) violation of the ISNT rule (i.e., normal eyes exhibit a characteristic configuration of neuroretinal rim thickness where: inferior > superior > nasal > temporal). Participants were considered to have OAG based on the modified International Society of Geographical and Epidemiological Ophthalmology criteria for the Korean population. The specific diagnostic criteria are as follows. Category 1 requires both (1) a reliable visual field defect consistent with glaucoma (fixation error and false positive error ≤ 1 and the presence of at least two locations of reduced sensitivity) and (2) glaucomatous optic disc (neuroretinal rim loss with VCDR or horizontal cup-disc ratio ≥ 0.6 or the presence of optic disc hemorrhage or the presence of retinal nerve fiber layer defects or asymmetry of VCDR ≥ 0.2). Category 2, if a visual field test is not available or is unreliable (fixation error or false positive error ≥ 2), requires VCDR ≥ 0.9 or asymmetry of VCDR ≥ 0.3 or the presence of retinal nerve fiber layer defects and violation of the ISNT rule. Category 3 criteria, requiring a visual acuity ≤ 3/60 and an IOP > 21 mmHg, was not included as diagnostic criteria for glaucoma in this study. Subjects with a shallow anterior chamber (peripheral anterior chamber depth ≤ 1/4 of the central corneal thickness) were excluded. From the OAG with normal IOP group, participants were further categorized into low-teen OAG (IOP < 15 mmHg) and high-teen OAG (IOP ≥ 15 mmHg) groups.

### Heavy metal levels

Blood lead and cadmium levels were measured using graphite furnace atomic absorption spectrometry (AAnalyst™ 600; Perkin-Elmer, Turku, Finland) and mercury levels were measured using the gold amalgamation method (DMA-80; Milestone, Sorisole, Italy). Blood samples were collected and stored in trace element ethylenediaminetetraacetic acid (EDTA) tubes (BD, Franklin Lakes, NJ, USA) for the heavy metal assays. The detection limits were 0.223, 0.087, and 0.05 μg/L for lead, cadmium, and mercury, respectively. The concentrations in all samples were higher than the detection limits. All blood analyses were carried out by the Seoul Medical Science Institute (SMSI) certified by the Korean Ministry of Health and Welfare. Internal and external quality control for this institute has been clearly described in a previous study [20].

### Other traits

Demographic variables included age group, sex, area of residence, occupation, income, and education level, which were acquired from the health interviews. Regarding region of residence, the 16 districts of South Korea were divided into two groups: 1) urban regions, including Seoul, Gyeonggi, Busan, Daegu, Incheon, Gwangju, Daejeoun, and Ulsan; and 2) rural regions, including Gangwon, Chungbuk, Chungnam, Jeonnam, Jeonbuk, Gyeongbuk, Gyeongnam, and Jeju. Occupation was classified into three groups: 1) white collar, comprising managers, professionals, clerical support workers, and service and sales workers; 2) blue collar, including agriculture, forestry, fishery, craft, and related trade workers, plant and machine operators and assemblers, and simple labor; and 3) inoccupation, including unemployed, retired, students, and homemakers. Income status was quantified by quartile. Finally, education level was categorized as elementary school or less, middle school graduate, high school graduate, or college graduate and beyond.

The other covariates analyzed in this study were health-related behaviors as follows: smoking (ever or never); exercise (days per week); body mass index (BMI), estimated as weight/height (kg/m^2^), categorized into two groups: 1) BMI < 25 kg/m^2^ and 2) BMI ≥ 25 kg/m^2^; and medical comorbidities, including diabetes mellitus, hypertension, hyperlipidemia, and anemia. Blood samples were collected from all subjects by venipuncture after fasting for at least 8 h. Specimens were transported immediately to the central laboratory (Neodin Medical Institute, Seoul, Korea), where all blood samples were analyzed within 24 h. Subjects were defined as having hypertension if they had a history of taking antihypertensive medication or when the measured systolic or diastolic blood pressure was ≥140 or ≥90 mmHg, respectively. Participants who had been diagnosed with diabetes by physicians or had a fasting glucose level ≥ 126 mg/dL were defined as having diabetes mellitus. Subjects were categorized as having anemia if their hemoglobin levels were <12 g/dL in non-pregnant women, <11d/gL in pregnant women, and <13 g/dL in men.

### Statistical analysis

Statistical analyses were performed using SAS statistical software (v. 9.3; SAS Institute, Inc., Cary, NC, USA) to account for the complex sampling design and provide nationally representative prevalence estimates. The PROC SURVEY procedures were used for each analysis, and the codes are briefly introduced in supplementary table (S1 Table). The demographic characteristics of the participants are presented as either means and standard error (SE) or proportions and SE. The Rao-Scott χ^2^ (for categorical variables) and analysis of variance (ANOVA) (for continuous variables) tests were used to compare participant characteristics and glaucoma diagnosis. Blood concentrations for lead, mercury, and cadmium were log-transformed because of their skewed distributions (P < 0.001; Kolmogorov-Smirnov test) (S2 Table), and geometric means and standard errors were compared using analysis of covariance after adjusting for age group, sex, region of residence, occupation, education level, smoking status, hypertension, family history of glaucoma, and intraocular pressure.

Multivariate logistic regression analyses were conducted to assess the association between the prevalence of glaucoma and blood heavy metal concentrations. For the logistic regression analyses, three models were established: model 1 used simple logistic regression; model 2 adjusted for age group and sex; and model 3 adjusted for model 2 and other potential confounding variables. Odds ratios (ORs) and 95% confidence intervals (CIs) for the prevalence of glaucoma were estimated for each heavy metal. In all analyses, P-values were two-tailed; P < 0.05 was considered to indicate statistical significance.

## Results

Of the 32,949 participants aged ≥19, 9,111 participants underwent measurement of blood heavy metal concentrations and ophthalmic examination. Among these participants, we excluded participants with following criteria: IOP > 21 mmHg (n = 20), shallow peripheral angle (n = 111), any signs of diabetic retinopathy or age-related macular degeneration (n = 1,043), history of any glaucoma medical treatment (n = 27), history of glaucoma, refractive or retinal surgery (n = 242), and missing values (n = 2,470). Finally, a total of 5,198 participants were included for the analyses. These participants included 4,989 non-glaucomatous subjects and 209 subjects with OAG with normal IOP. Based on IOP level, the OAG with normal IOP group was divided into low-teen (n = 136) and high-teen (n = 73) OAG groups. The demographic characteristics according to the glaucoma diagnosis are summarized in Table 1. OAG with normal IOP subjects were older (P < 0.001), predominantly male (P = 0.009), less educated (P = 0.043), had a higher proportion of having ever smoked (P = 0.035), had a higher proportion of having hypertension (P < 0.001), and had higher levels of IOP (P = 0.044) than those without the disease.

**Table 1:** Demographics and general health characteristics of the normal and open-angle glaucoma (OAG) with normal intraocular pressure (IOP) populations.

Parameter	Normal (n = 4,989)	OAG with normal IOP (n = 209)			P^†^
			Low-teen OAG (n = 136)	High-teen OAG (n = 73)	
Demographics					
Age groups					<0.001*
19–29	10.0 (0.4)	2.9 (1.3)*	5.0 (2.2)*	0 (0)*	
30–39	11.0 (0.4)	9.5 (2.3)	8.0 (2.8)	11.8 (4.3)	
40–49	33.6 (0.7)	24.9 (3.8)	17.8 (4.0)	35.3 (7.0)	
50–59	25.0 (0.6)	26.7 (3.6)	29.1 (4.8)	23.1 (5.8)	
60–69	11.6 (0.4)	15.1 (2.4)	16.8 (3.2)	12.6 (4.0)	
≥70	8.8 (0.8)	20.9 (4.9)	23.3 (6.5)	17.2 (7.0)	
Female, %	51.9 (0.6)	39.2 (4.6)*	40.3 (6.0)*	34.9 (7.5)*	0.009*
Area of residence, %					0.540
Urban region	68.0 (1.0)	70.8 (4.4)	64.6 (5.7)	79.7 (5.6)	
Rural region	32.0 (1.0)	29.2 (4.4)	35.4 (5.7)	20.3 (5.6)	
Education, %					0.043*
≤Elementary school	22.1 (0.7)	30.9 (4.7)*	34.7 (6.4)	25.6 (7.1)	
Middle school graduate	13.1 (0.6)	14.0 (3.0)	12.8 (3.8)	15.8 (4.8)	
High school graduate	39.0 (0.9)	29.8 (3.9)	29.5 (5.0)	30.3 (6.8)	
≥College graduate	25.8 (0.8)	25.2 (3.5)	23.0 (4.2)	28.3 (5.8)	
Occupation, %					0.273
White collar	35.6 (0.8)	30.0 (3.9)	29.1 (5.0)*	31.3 (6.5)	
Blue collar	30.6 (0.9)	29.8 (4.1)	23.2 (4.6)	39.1 (7.1)	
Inoccupation	33.8 (0.9)	40.2 (4.7)	47.7 (6.1)	29.6 (7.3)	
Income, %					0.369
Quartile 1	25.9 (0.8)	25.5 (3.9)	25.1 (5.2)	26.1 (6.3)	
Quartile 2	26.0 (0.8)	19.4 (3.6)	14.9 (3.3)	25.8 (6.9)	
Quartile 3	25.1 (0.8)	28.2 (4.5)	28.1 (6.0)	28.2 (6.3)	
Quartile 4	22.9 (0.8)	27.0 (3.9)	31.9 (5.4)	19.9 (5.7)	
Smoking status, %					0.035*
Ever	44.8 (0.8)	55.3 (4.9)*	54.5 (6.0)	56.5 (7.6)	
Never	55.2 (0.8)	44.7 (4.9)	45.5 (5.7)	43.5 (7.6)	
Exercise days per week, %					0.308
0	63.7 (0.9)	68.1 (4.3)	70.7 (5.0)	64.3 (6.9)	
1	11.0 (0.6)	10.3 (2.8)	8.9 (3.5)	12.4 (4.5)	
2	7.8 (0.5)	7.9 (2.3)	7.1 (2.5)	9.1 (4.1)	
3	7.2 (0.5)	4.1 (1.5)	6.2 (2.3)	1.1 (0.9)	
≥4	10.3 (0.5)	9.5 (2.5)	7.1 (2.8)	13.1 (4.7)	
BMI, %					0.415
<25 kg/m^2^	69.0 (0.7)	72.7 (4.2)	71.2 (5.6)	72.2 (5.2)	
≥25 kg/m^2^	31.0 (0.7)	28.1 (3.4)	28.3 (4.6)	27.8 (5.2)	
Medical comorbidities, %					
Diabetes mellitus	7.5 (0.5)	10.4 (2.9)	12.6 (4.0)	7.2 (3.9)	0.263
Hypertension	18.6 (0.7)	34.5 (4.3)*	38.9 (6.1)*	28.1 (6.1)	<0.001*
Hyperlipidemia	14.0 (0.6)	17.5 (3.4)	16.6 (4.2)	18.8 (5.7)	0.275
Anemia	8.1 (0.5)	13.6 (4.0)	11.2 (4.6)	17.1 (7.2)	0.090
Laboratory tests					
Fasting glucose, mg/dL	96.9 (0.3)	98.3 (1.9)	97.1 (1.9)	100.1 (3.8)	0.459
Ferritin, ng/mL	89.5 (1.9)	126.0 (26.3)	147.6 (43.5)	94.5 (9.4)	0.165
Aspartate aminotransferase, U/L	22.8 (0.2)	24.7 (1.6)	25.8 (2.4)	23.1 (1.3)	0.067
Alanine transaminase, U/L	22.6 (0.3)	23.1 (1.3)	23.5 (1.6)	22.6 (1.9)	0.681
Refractive status^‡^					0.053
Emmetropia	54.5 (0.9)	43.9 (4.7)	47.2 (5.9)	39.1 (7.1)*	
Myopia					
Mild	21.8 (0.7)	19.0 (3.3)	16.5 (3.9)	22.7 (6.0)	
Moderate	8.4 (0.5)	15.0 (3.1)	13.0 (3.3)	17.9 (5.1)	
Severe	4.5 (0.4)	7.8 (2.1)	5.2 (2.1)	11.6 (4.4)	
Hyperopia	10.8 (0.6)	14.3 (3.2)	18.1 (4.6)	8.7 (4.3)	
Family history of glaucoma	1.8 (0.2)	2.0 (1.1)	2.1 (1.5)	1.8 (1.8)	0.846
IOP, mmHg	13.8 (0.1)	14.6 (0.3)*	12.4 (0.2)*	17.7 (0.3)*	0.044*

All data are expressed as weighted means or weighted frequency with standard error (SE).

IOP, intraocular pressure; OAG, open-angle glaucoma; BMI, body mass index.

*P < 0.05 versus the normal control group.

^†^P value between normal and OAG with normal IOP group. Rao-Scott χ^2^ (for categorical variables) or analysis of variance (ANOVA) (for continuous variables) tests were used.

Table 2 shows different demographic characteristics according to gender. Only age group and presence of hypertension were significantly associated with OAG with normal IOP in both men (P = 0.016 and P < 0.001, respectively) and women (P < 0.001 and P = 0.022, respectively).

**Table 2:** Demographics and general health characteristics of normal and OAG with the normal IOP population by gender.

Parameter	Men (n = 2,573)			Women (n = 2,625)		
	Normal (n = 2,440)	OAG with normal IOP (n = 133)	P^†^	Normal (n = 2,549)(n = 2,549)	OAG with normal IOP (n = 76)	P^†^
Demographics						
Age groups			0.016*			<0.001*
19–29	10.7 (0.6)	3.8 (2.1)*		9.3 (0.5)	1.7 (1.0)	
30–39	11.5 (0.6)	7.6 (2.6)		10.5 (0.5)	12.5 (4.5)	
40–49	35.0 (0.9)	32.9 (5.3)		32.3 (0.9)	12.4 (4.2)	
50–59	24.8 (0.8)	30.6 (4.6)		25.2 (0.8)	20.7 (5.9)	
60–69	11.4 (0.6)	14.3 (3.0)		11.8 (0.6)	16.3 (4.3)	
≥70	6.6 (0.8)	10.9 (3.8)		11.0 (1.2)	36.3 (9.3)	
Area of residence, %			0.110			0.472
Urban region	67.4 (1.2)	75.9 (4.7)		68.4 (1.2)	62.9 (7.9)	
Rural region	32.6 (1.2)	24.1 (4.7)		31.6 (1.2)	37.1 (7.9)	
Occupation, %			0.991			0.001*
White collar	39.9 (1.2)	39.3 (5.1)		31.7 (1.1)	15.9 (5.4)	
Blue collar	40.5 (1.3)	41.2 (5.4)		21.4 (1.2)	12.3 (4.2)	
Inoccupation	19.6 (1.0)	19.5 (4.5)		46.9 (1.3)	71.8 (6.8)	
Education, %			0.791			<0.001*
≤Elementary school	14.2 (0.9)	11.7 (3.4)		29.5 (1.2)	60.3 (7.7)*	
Middle school graduate	12.5 (0.8)	15.6 (4.1)		13.6 (0.8)	11.6 (4.1)	
High school graduate	41.5 (1.2)	41.5 (5.2)		36.6 (1.2)	12.0 (3.9)	
≥College graduate	31.8 (1.2)	31.2 (4.3)		20.3 (0.9)	16.0 (5.3)	
Smoking status, %			0.473			0.667
Ever	82.5 (0.9)	85.6 (3.9)		9.7 (0.8)	7.8 (4.0)	
Never	17.5 (0.8)	14.4 (3.9)		90.3 (0.8)	92.2 (4.0)	
Medical comorbidities, %						
Hypertension	17.4 (0.9)	33.4 (4.9)*	<0.001	19.7 (1.1)	36.2 (8.4)*	0.022*
Family history of glaucoma	1.9 (0.3)	1.4 (1.3)	0.763	1.65 (0.3)	2.84 (2.1)	0.475
IOP, mmHg	14.1 (0.1)	14.7 (0.4)	0.132	13.7 (0.07)	14.4 (0.6)	0.282

All data are expressed as weighted means or weighted frequency with SE.

OAG, open-angle glaucoma; IOP, intraocular pressure.

*P < 0.05 versus the normal control group.

^†^Rao-Scott χ^2^ (for categorical variables) or ANOVA (for continuous variables) tests were used.

When the geometric means of blood lead, mercury, and cadmium concentrations adjusted for age group, sex, region of residence, occupation, education level, smoking status, hypertension, family history of glaucoma, and intraocular pressure.were compared between the non-glaucomatous and OAG with normal IOP groups, non-significant differences were noted for all three heavy metal levels (P = 0.671, P = 0.237, and P = 0.074, respectively; Table 3). When the OAG with normal IOP group was divided into low-teen and high-teen OAG groups, only the blood cadmium level was significantly higher in subjects with low-teen OAG than in the non-glaucomatous group (P = 0.028), whereas no significant differences in any of the three heavy metal levels were observed in the high-teen OAG group. In men, the significant difference between the normal and OAG with normal IOP groups was only observed for blood cadmium levels (P = 0.014), and the significant difference was also observed for low-teen OAG in subgroup analysis (P = 0.014). In women, on the other hand, the difference was not significant for all three heavy metals.

**Table 3:** Blood heavy metal concentrations in study subjects according to glaucoma diagnosis adjusted for confounding factors.

Heavy metal	Normal	OAG with normal IOP			*P*^†^
			Low-teen OAG	High-teen OAG	
**Total**	**n = 4,989**	**n = 209**	**n = 136**	**n = 73**		
Lead (μg/dL)	2.32 (2.28–2.35)	2.28 (2.13–2.44)	2.38 (2.18–2.60)	2.13 (1.92–2.37)	0.671	
Mercury (μg/L)	4.10 (4.00–4.20)	3.79 (3.34–4.30)	3.96 (3.33–4.71)	3.54 (2.97–4.22)	0.237	
Cadmium (μg/dL)	1.05 (1.03–1.07)	1.15 (1.04–1.27)	1.18 (1.06–1.30)*	1.11 (0.92–1.33)	0.074	
**Men**	**n = 2,440**	**n = 133**	**n = 79**	**n = 54**		
Lead (μg/dL)	2.75 (2.70–2.80)	2.69 (2.52–2.87)	2.78 (2.60–2.97)	2.57 (2.28–2.91)	0.501	
Mercury (μg/L)	4.99 (4.83–5.14)	5.03 (4.47–5.66)	5.41 (4.52–6.47)	4.58 (3.90–5.39)	0.891	
Cadmium (μg/dL)	0.94 (0.92–0.97)	1.10 (0.97–1.25)*	1.12 (0.98–1.28)*	1.07 (0.87–1.33)	0.014*	
**Women**	**n = 2,549**	**n = 76**	**n = 57**	**n = 19**		
Lead (μg/dL)	1.97 (1.93–2.00)	1.96 (1.71–2.25)	2.10 (1.75–2.51)	1.72 (1.43–2.06)	0.940	
Mercury (μg/L)	3.39 (3.29–3.51)	2.75 (2.16–3.48)	2.81 (2.07–3.83)	2.58 (1.80–3.73)	0.078	
Cadmium (μg/dL)	1.16 (1.13–1.19)	1.17 (1.01–1.34)	1.20 (1.04–1.38)	1.10 (0.81–1.49)	0.931	

IOP, intraocular pressure; OAG, open-angle glaucoma.

Expressed as geometric mean (95% CI).

*P<0.05 compared to normal control group.

^†^P value between normal and OAG with normal IOP group.

Adjusted for age group, sex, region of residence, occupation, education level, smoking status, hypertension, family history of glaucoma, and intraocular pressure.

The associations between the log-transformed levels of the three heavy metals and prevalence of OAG with normal IOP from multiple logistic regression analyses are shown in Table 4. After adjusting for potential confounding factors, lead and mercury did not show any significant associations with OAG with normal IOP (P = 0.701 and P = 0.239, respectively), as well as low-teen (P = 0.517 and P = 0.769, respectively) and high-teen OAG (P = 0.157 and P = 0.073, respectively). In contrast, log-transformed cadmium levels were positively associated only with prevalence of low-teen OAG (OR, 1.41; 95% CI, 1.03–1.93; P = 0.026), while not showing any significant association with OAG with normal IOP (OR, 1.33; 95% CI, 0.97–1.83; P = 0.073) or high-teen OAG (OR, 1.16; 95% CI, 0.67–2.00; P = 0.592).

**Table 4:** Odds ratios (ORs) for the prevalence of glaucoma according to log-transformed blood lead, mercury, and cadmium levels.

	Lead			Mercury			Cadmium		
	OR	95% CI	P	OR	95% CI	P	OR	95% CI	P
OAG with normal IOP									
Model 1^†^	1.25	0.91, 1.74	0.174	0.92	0.69, 1.21	0.536	1.37	1.07, 1.75	0.012*
Model 2^‡^	0.93	0.66, 1.33	0.696	0.86	0.67, 1.11	0.236	1.30	0.96, 1.76	0.085
Model 3^§^	0.93	0.65, 1.34	0.701	0.86	0.66, 1.11	0.239	1.33	0.97, 1.83	0.073
Low-teen OAG									
Model 1^†^	1.41	0.94, 2.11	0.097	0.95	0.65, 1.37	0.768	1.64	1.31, 2.05	0.001*
Model 2^‡^	1.12	0.72, 1.76	0.616	0.93	0.67, 1.29	0.666	1.45	1.06, 1.97	0.018*
Model 3^§^	1.16	0.74, 1.83	0.517	0.95	0.67, 1.35	0.769	1.41	1.03, 1.93	0.026*
High-teen OAG									
Model 1^†^	1.06	0.63, 1.78	0.831	0.87	0.55, 1.38	0.549	1.37	0.96, 1.97	0.391
Model 2^‡^	0.70	0.41, 1.20	0.192	0.76	0.52, 1.11	0.156	1.12	0.67, 1.87	0.667
Model 3^§^	0.65	0.36, 1.18	0.157	0.74	0.53, 1.03	0.073	1.16	0.67, 2.00	0.592

IOP, intraocular pressure; OAG, open-angle glaucoma; CI, confidence interval.

*P < 0.05.

^†^Model 1: crude OR.

^‡^Model 2: adjusted for age group and sex.

^§^Model 3: adjusted for age group, sex, region of residence, occupation, education level, smoking status, hypertension, family history of glaucoma, and IOP.

Next, we investigated gender differences in the association between prevalence of OAG with normal IOP and the log-transformed blood cadmium levels. Multivariate logistic regression analyses revealed that only log-transformed cadmium levels were positively associated with OAG with normal IOP in fully adjusted model (OR, 1.61; 95% CI, 1.11–2.34; P = 0.013) in men, whereas we did not find a significant relationship between blood levels of any of the three heavy metals and OAG with normal IOP in women (Table 5). Similar results were observed in the subtype analyses with low-teen OAG, where positive association with only log-transformed cadmium levels was observed (OR, 1.65; 95% CI, 1.10–2.48; P = 0.016) in men, while no significant association was found in women for all three heavy metals. In contrast, high-teen OAG did not show significant associations with log-transformed heavy metal levels in either sex.

**Table 5:** Gender differences in ORs for a glaucoma diagnosis according to log-transformed blood lead, mercury, and cadmium levels.

	Lead			Mercury			Cadmium		
	OR	95% CI	P	OR	95% CI	P	OR	95% CI	P
**Men**									
OAG with normal IOP									
Model 1^†^	1.06	0.75, 1.51	0.750	1.05	0.83, 1.32	0.689	1.68	1.24, 2.26	0.001*
Model 2^‡^	0.91	0.63, 1.32	0.619	1.04	0.83, 1.30	0.764	1.55	1.12, 2.16	0.009*
Model 3^§^	0.87	0.59, 1.29	0.493	1.03	0.81, 1.30	0.842	1.61	1.11, 2.34	0.013*
Low-teen OAG									
Model 1^†^	1.15	0.79, 1.68	0.456	1.10	0.79, 1.54	0.584	1.71	1.19, 2.47	0.004*
Model 2^‡^	1.10	0.69, 1.48	0.962	1.16	0.81, 1.53	0.497	1.61	1.10, 2.34	0.014*
Model 3^§^	1.00	0.65, 1.54	0.988	1.19	0.84, 1.70	0.323	1.65	1.10, 2.48	0.016*
High-teen OAG									
Model 1^†^	0.95	0.50, 1.79	0.862	0.98	0.73, 1.32	0.917	1.64	0.99, 2.71	0.056
Model 2^‡^	0.80	0.41, 1.57	0.511	0.93	0.68, 1.27	0.650	1.49	0.83, 2.66	0.179
Model 3^§^	0.66	0.29, 1.50	0.325	0.87	0.61, 1.24	0.442	1.41	0.84, 2.38	0.194
**Women**									
OAG with normal IOP									
Model 1^†^	1.10	0.57, 2.09	0.783	0.58	0.33, 1.04	0.067	1.19	0.80, 1.75	0.391
Model 2^‡^	0.98	0.52, 1.87	0.952	0.62	0.35, 1.11	0.107	0.97	0.58, 1.62	0.913
Model 3^§^	0.99	0.53, 1.84	0.970	0.63	0.37, 1.08	0.091	1.00	0.60, 1.65	0.991
Low-teen OAG									
Model 1^†^	1.49	0.62, 3.55	0.373	0.56	0.25, 1.26	0.162	1.47	0.98, 2.19	0.060
Model 2^‡^	1.32	0.55, 3.20	0.533	0.67	0.32, 1.38	0.279	1.20	0.72, 2.00	0.479
Model 3^§^	1.44	0.67, 3.08	0.353	0.64	0.31, 1.32	0.225	1.10	0.66, 1.85	0.709
High-teen OAG									
Model 1^†^	0.65	0.29, 1.45	0.289	0.36	0.12, 1.12	0.080	0.85	0.45, 1.61	0.621
Model 2^‡^	0.57	0.26, 1.25	0.161	0.42	0.16, 1.12	0.084	0.68	0.27, 1.72	0.412
Model 3^§^	0.62	0.23, 1.07	0.078	0.66	0.32, 1.39	0.276	1.07	0.47, 2.43	0.877

IOP, intraocular pressure; OAG, open-angle glaucoma; CI, confidence interval.

*P < 0.05.

^†^Model 1: crude OR.

^‡^Model 2: adjusted for age group.

^§^Model 3: adjusted for age group, region of residence, occupation, education level, smoking status, hypertension, family history of glaucoma, and IOP.

## Discussion

This study investigated the association between three well-known toxic heavy metals (lead, mercury, and cadmium) and the prevalence of OAG with normal IOP, low-teen OAG, and high-teen OAG. We found that blood cadmium levels were associated with low-teen OAG, while no associations were found for lead or mercury. In addition, we found gender differences in the association between heavy metals and OAG with normal baseline IOP, in which the positive association was shown for men but not for women.

Cadmium is a non-essential, highly toxic metabolic element that can be found naturally in the surrounding environment. Due to rapid industrialization, there is an increasing risk of cadmium exposure from occupational and environmental sources, mainly through dietary intake or inhalation of polluted air. Cadmium toxicity in the human body has been widely investigated. It can act as an inflammatory agent, causing oxidative stress to cells, such as neurons, retinal cells, renal tubules, and vascular endothelial cells [36, 37]. Moreover, the vascular system is a critical target of cadmium toxicity, in which cadmium causes vascular remodeling and stiffening [37–39], leading to an increased risk of various systemic vascular diseases, including atherosclerosis [40], myocardial infarction [18], peripheral arterial diseases [41], and hypertension [11].

In this study, blood cadmium levels were positively associated with prevalence of low-teen OAG, but not whole OAG with normal IOP or high-teen OAG, after adjusting for age group, sex, region of residence, occupation, education level, smoking status, hypertension, and intraocular pressure. The results from previous studies have indicated that different mechanisms may exist among NTG patients, and non-pressure factors might contribute to glaucoma development, especially in low-teen OAG subjects. In particular, recent studies suggested that vascular factors may play a more significant role in low-teen OAG by demonstrating more central retinal nerve fiber layer defect patterns [6], more close relationship with disc hemorrhage in progression [9], and less frequency of simultaneous venous pulsation observed in subjects with low-teen OAG when compared to those with high-teen OAG [8]. More recently, using KNHANES data, we demonstrated that low-teen OAG subjects were more closely associated with hypertension, especially those taking antihypertensive medicine [42]. Based on previous studies and the results of this study, we suggest that cadmium might influence the development of NTG via its vascular toxicity, particularly in patients with low baseline IOP. Another possible mechanism that could contribute to glaucomatous damage is direct toxicity to retinal ganglion cells, since a relationship between cadmium and neurodegenerative diseases, such as Alzheimer’s and Parkinson’s diseases, has been identified [19]. However, there is still minimal evidence to support that cadmium has causative effect on the development of glaucomatous optic nerve damage, and further investigations are needed to reveal the exact toxic mechanism of cadmium.

In general, women are known to have higher cadmium levels than men under similar exposure conditions due to the higher prevalence of iron deficiency and hormonal effects after menopause [34]. However, regarding the association between cadmium and OAG, higher cadmium levels were significantly associated with the prevalence of whole OAG with normal IOP and low-teen OAG only in men, not in women. Although no previous studies have demonstrated differential effects of cadmium on glaucomatous optic neuropathy by gender, several lines of evidence suggest that cadmium toxicity might differ between men and women. Consistent with our findings, using data from KNHANES, previous studies demonstrated that high blood cadmium levels were associated with AMD prevalence [20], metabolic syndrome [16], and chronic obstructive pulmonary disease [43] only in Korean men. Such gender difference may be partly explained by different exposure routes of cadmium in men and women. Occupational exposure, such as the metal and mining industry, transportation, and repair services, could be important for men, whereas cadmium ingestion from dietary sources could be the main exposure route for women [44]. These differences in exposure sources could result in different effective doses, since the percentage of cadmium absorption through the respiratory tract is 10–50%, approximately twice that of the gastrointestinal tract (5–20%) [44]. In addition, different metabolic pathways of respiratory and dietary exposure could influence the severity of toxicity in body tissues. Smoking, which was significantly higher in men, might also affect the gender difference. However, in this study, the difference persisted even after adjusting for smoking status, indicating that smoking does not explain the gender difference.

Although no significant associations between blood levels of lead or mercury and glaucoma prevalence were found in the current study, toxicity from exposure to lead and mercury has been demonstrated in many studies. Especially, lead has been suggested as an associated factor for glaucoma. Yuki et al. [28] demonstrated that hair lead levels were significantly higher in women with OAG with low-teen baseline IOP compared with the normal control, while Ekinci et al. [29] reported that chronic occupational exposure of lead might lead to decreased retinal nerve fiber layer thickness. Moreover, mercury is a highly toxic metallic element, and several studies have revealed toxic effects on eye and visual function. In vivo studies showed retinal toxicity of mercury [45, 46], especially to the photoreceptor layer, and other clinical studies reported visual dysfunction caused by mercury intoxication [47, 48].

Recently, Lin et al. [49] published a paper regarding the association between trace heavy metal levels and glaucoma prevalence using data from KNHANES 2008 and 2009. They revealed that blood manganese levels were negatively associated with glaucoma prevalence, while mercury had a marginally significant association with glaucoma. Conversely, we did not observe an association between blood mercury and prevalence of OAG with normal IOP. One possible reason for such discordant results is that we used log-transformed blood heavy metal levels because of the skewed distribution, while the previous study used the raw blood heavy metal level data for the analyses. Another possible reason is that the inclusion and exclusion criteria were different and the KNHANES data included for the analyses differed. These factors may have influenced the statistical analyses, leading to inconsistent results between the studies.

Several limitations should be considered when interpreting the results of this study. First, the baseline IOP measurement was performed only once, which might not have been sufficient to accurately evaluate the IOP level of each participant and define the glaucoma type. Therefore, we used term ‘OAG with normal IOP’ instead of using NTG in this study. However, most of the OAG patients with normal IOP levels in this study may be representative of NTG patients since large number of subjects was included for the analyses. Second, heavy metal exposure was evaluated using only blood samples, not bone or soft tissue samples. As a result, the measurements might not accurately reflect chronic exposure. However, it is generally accepted that blood concentrations of heavy metals can reflect daily exposure status and can be used as an indicator of the body burden of heavy metals from low environmental exposure [50]. Third, a causal relationship between heavy metal levels and OAG cannot be identified from the results of this study because of the cross-sectional study design. Further experimental and clinical studies are necessary to demonstrate the specific association. Fourth, the size of the groups, normal and OAG with normal IOP, were uneuqual, and only a subpopulation of participants underwent blood heavy metal level investigations, which may cause statistical bias. In addition, onset and duration of glaucoma suffering is not provided in the survey, which may be another important confounding factor. These limitations should be taken into consideration when interpreting the results of our study.

Overall, the results from this study suggest that higher blood cadmium levels may be associated with low-teen OAG, especially in men. Still, differences in associations with heavy metals for each OAG group hinder us to make definite conclusion on causal links between heavy metals and OAG. Future investigations are warranted to reveal and confirm the exact toxic mechanism how heavy metals affect the development of glaucomatous optic nerve damage. Regardless, our results further add to the previous idea that non-pressure factors may be more contributable to the pathogenesis of low-teen OAG than to that of high-teen OAG.

## Supporting Information

S1 Table SAS codes for statistical analyses. (DOCX)

S2 Table Distribution of blood heavy metals among study subjects. (DOCX)
