# Supplementary material for: Three Toxic Heavy Metals in Open-Angle Glaucoma with Low-Teen and High-Teen Intraocular Pressure: A Cross-Sectional Study from South Korea
Source: PLoS One. 2016 Oct 21;11(10):e0164983. doi: 10.1371/journal.pone.0164983 (PMC5074541; doi:10.1371/journal.pone.0164983)
Supplement: S1 Table — (DOCX) [file pone.0164983.s001.docx]

| **Statistical analyses** | **Codes** |
| --- | --- |
| Rao-Scott χ^2^ test | PROC SURVEYFREQ DATA = Analysis_data NOMCAR;  STRATA kstrata;  CLUSTER psu;  WEIGHT 4_year_weight;  TABLES Glaucoma_diagnosis*confounding_factors / CL ROW CLWT CHISQ WCHISQ;  RUN; |
| Analysis of variance (ANOVA) | PROC SURVEYREG DATA= Analysis_data NOMCAR;  STRATA kstrata;  CLUSTER psu;  WEIGHT 4_year_weight;  DOMAIN age_above_19;  CLASS Glaucoma_diagnosis;  MODEL Confounding_factors=Glaucoma_diagnosis /NOINT CLPARM SOLUTION VADJUST=none;  RUN; |
| Logistic regression analyses | PROC SURVEYLOGISTIC DATA= Analysis_data NOMCAR;  STRATA kstrata;  CLUSTER psu;  WEIGHT 4_year_weight;  DOMAIN age_above_19;  CLASS age_group (REF= '19-29 yrs' ) sex (REF='Male') region (REF='Rural') occupation (REF='Inoccupation') smoking_status (PARAM=REF REF='Never') hypertension (REF='Normal') education (REF='Elementary school or less') family_history (REF='No history') /param=ref;  MODEL Glaucoma_diagnosis (event='1')=heavy_metal age_group sex region occupation intraocular_pressure smoking_status hypertension education family_history /VADJUST=none;  RUN; |

**Supplementary Table 1. SAS codes for statistical analyses.**
