# Supplementary material for: Three Toxic Heavy Metals in Open-Angle Glaucoma with Low-Teen and High-Teen Intraocular Pressure: A Cross-Sectional Study from South Korea
Source: PLoS One. 2016 Oct 21;11(10):e0164983. doi: 10.1371/journal.pone.0164983 (PMC5074541; doi:10.1371/journal.pone.0164983)
Supplement: S2 Table — (DOCX) [file pone.0164983.s002.docx]

**S2 Table.**

| **Heavy metal** | **Mean** | **Median** | **SD** | **Skewness** | **Kolmogorov-Smirnov**  **(P)** |
| --- | --- | --- | --- | --- | --- |
| Lead (µg/dL) | 2.31 | 2.10 | 1.24 | 4.97 | <0.01 |
| Mercury (µg/L) | 4.45 | 3.46 | 3.92 | 9.52 | <0.01 |
| Cadmium (µg/dL) | 1.08 | 0.95 | 0.69 | 1.64 | <0.01 |

SD, standard deviation.
